# Ultra-fast extubation following cardiac surgery improves hemodynamic stability and reduces ICU workload

**DOI:** 10.3389/fcvm.2025.1695955

**Published:** 2025-11-24

**Authors:** Vito Angiuli, Marc Rohner, Maria Wittmann, Florian Piekarski, Jacqueline Kruse, Miriam Silaschi, Andrea Kunsorg, Andreas Mayr, Izdar Abulizi, Jan Speller, Farhad Bakhtiary, Markus Velten

**Affiliations:** 1 Department of Anesthesiology and Intensive Care, University Hospital Bonn, Bonn, Germany; 2 Department of Cardiac Surgery, University Hospital Bonn, Bonn, Germany; 3 Institute for Medical Biometry and Statistics, Marburg University, Marburg, Germany; 4 Institute for Informatics, University of Münster, Muenster, Germany; 5 Department of Anesthesiology and Pain Management, The University of Texas Southwestern Medical Center, Dallas, TX, United States

**Keywords:** enhanced recovery after cardiac surgery, ultrafast extubation, extubation in the OR, ICU workload, cardiac anesthesia

## Abstract

**Objective:**

To address ICU capacity constraints, particularly during the COVID-19 pandemic, we implemented an ultrafast extubation (UFE) protocol involving operating room extubation (ORE) following on-pump cardiac surgery. We hypothesize that ORE is safe, improves postoperative outcomes, and reduces ICU workload compared with standard extubation in the ICU (ICUE).

**Methods:**

We retrospectively analyzed 397 adult patients who underwent on-pump cardiac surgery at a tertiary center between February and October 2021. Following propensity score matching (*n* = 224), patients were stratified into ORE and ICUE groups. Primary outcomes included hemodynamic stability assessed via the simplified acute physiology score (SAPS) and ICU workload measured by the Therapeutic Intervention Scoring System (TISS). Secondary outcomes included duration of mechanical ventilation, vasopressor requirements, ICU and hospital length of stay (LOS), and transfusion needs.

**Results:**

Patients extubated in the OR demonstrated significantly lower SAPS and TISS scores on admission and cumulatively during ICU treatment (*p* < 0.001), reflecting improved hemodynamic stability and reduced ICU workload. ORE patients also had lower postoperative vasoactive–inotropic scores and reduced catecholamine use (all *p* < 0.001). ICU LOS was significantly shorter in the ORE group (median 24.5 vs. 45.0 h, *p* = 0.023), while hospital LOS was comparable.

**Conclusion:**

Ultrafast extubation after cardiac surgery appears to be a safe and effective strategy to reduce ICU workload and resource use without compromising patient outcomes. ORE may be a valuable component of enhanced recovery protocols in cardiac surgical care.

## 1 Introduction

Enhanced recovery after surgery programs, including specialized perioperative anesthetic management protocols, have progressively evolved over the past decade. For patients undergoing cardiac surgery, these protocols are designed to minimize ventilation duration, shorten intensive care unit (ICU) treatment, and reduce overall hospital length of stay (LOS), ultimately improving patient outcomes, enhancing resource utilization, and promoting efficient and cost-effective care (1, 2).

**Figure 1:**
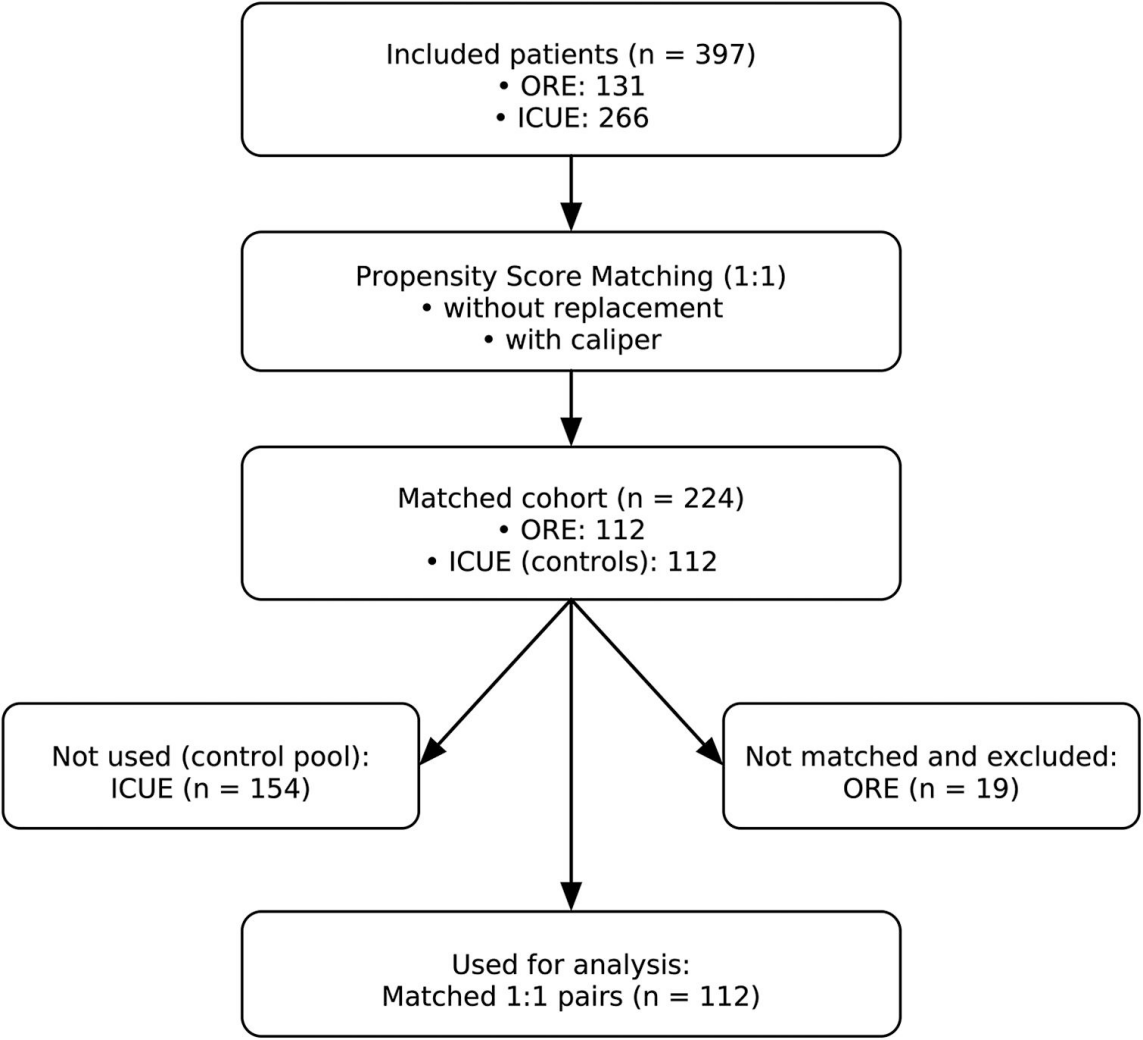
Study flow diagram. From 397 eligible patients (ORE, *n* = 131; ICUE, *n* = 266), 1:1 propensity score matching (without replacement, with caliper) resulted in 224 patients in the matched cohort (ORE, *n* = 112; ICUE, *n* = 112). Unmatched patients (ICUE, *n* = 154; ORE, *n* = 19) were excluded. The final analysis included 112 matched pairs.

ICU capacity constraints due to staffing shortages impact up to 76% of all intensive care units in Germany, leaving as many as 22% of ICU beds unavailable, mainly due to a lack of nursing staff (3). These constraints result in delays of procedures that require postsurgical intensive care (3). Such delays can lead to disease progression, making surgeries more invasive, complex, and risky, and often result in suboptimal outcomes. This is particularly critical in cardiac surgery, where postponement increases disease severity, surgical complexity, and ICU length of stay, further straining capacity and delaying care for other patients. Securing ICU resources for surgical cases is essential to prevent this cycle and minimize risk for treatment delays.

Each year, over 100,000 cardiac surgical procedures are performed in Germany (4). While the predicted risk profiles of these patients have increased, in-hospital mortality in cardiac surgery remains low, ranging between 2.7 and 3.7%, depending on the procedure type (5). However, postoperative morbidity remains a significant concern, with complications such as myocardial infarction (up to 22%), postoperative delirium (up to 50%), and acute kidney injury (AKI) (up to 40%). These complications increase the duration of ICU treatment and healthcare costs (6). The rising complexity of care is further compounded by an aging population; over 50% of cardiac surgery patients in Germany are over 70 years old (4). This demographic often presents with multiple comorbidities, including hypertension, atrial fibrillation, heart failure, renal insufficiency, neurocognitive dysfunction, and frailty, an independent predictor of increased surgical mortality (7).

Given the limited healthcare resources, a more efficient utilization of ICU facilities is essential to address the growing demands and escalating costs associated with cardiac surgery. An “enhanced recovery after cardiac surgery” program (ERACS) offers a potential solution (8). Key components of ERACS include early postoperative extubation, multimodal pain management, presurgical optimization (e.g., patient blood management), risk stratification, and routine delirium screening. These measures aim to shorten ICU stays, reduce postoperative complications, and improve overall outcomes without compromising patient safety.

Economic feasibility is a critical consideration when implementing new medical strategies. Enhanced recovery programs must demonstrate cost-effectiveness while maintaining or improving health outcomes. ERACS principles, now widely adopted across various surgical specialties, have redefined perioperative care by improving efficiency and patient outcomes. However, evidence for ERACS in cardiac surgery remains limited, with only a few single-center studies suggesting potential benefits for perioperative enhanced recovery interventions (9–11).

We hypothesize that implementing an ultrafast operating room extubation (ORE) protocol improves postoperative hemodynamic stability and reduces ICU workload compared with standard ICU extubation (ICUE), without increasing adverse events. To test this hypothesis, we evaluated hemodynamic stability by the simplified acute physiology score (SAPS) and ICU workload by the Therapeutic Intervention Scoring System (TISS). Secondary outcomes included length of stay.

## 2 Materials and methods

### 2.1 Study design

After study approval by the Ethics Committee of the University of Bonn (No.: 107/20, date of approval 18 March 2020), in accordance with §15 of the Medical Association Nordrhein’s professional code of conduct and the Declaration of Helsinki, we retrospectively reviewed all on-pump cardiac surgery cases performed at the University Medical Center Bonn, Germany, between February and October 2021. Data were extracted from digital medical records and the patient data management system (PDMS; Dräger). Inclusion criteria were age >18 years and cardiopulmonary bypass (CPB). Exclusion criteria included aortic arch reconstruction, already ventilated patients, re-surgery, and insufficient or missing relevant data. A total of 397 cases were identified and used for subsequent analyses. The primary endpoint of this study was hemodynamic stability assessed by the simplified acute physiology score (SAPS) and ICU workload measured by the Therapeutic Intervention Scoring System (TISS). Secondary endpoints comprised the total hospital and ICU length of stay (LOS), the duration of mechanical ventilation, and various clinical parameters, including catecholamine requirements.

### 2.2 Anesthesia protocol

Upon arrival in the induction suite and after a WHO-recommended security check, pulse oximetry, electrocardiogram (ECG), and a peripheral i.v. line were established. Under local anesthesia, the left radial artery was cannulated for blood pressure measurements and blood gas analyses. An INVOS™ 5100C Medtronic, Inc. (Minneapolis, MN, USA) probe was attached to the forehead for continuous monitoring of cerebral oxygenation saturation (rSO_2_) using near infrared spectroscopy (NIRS). A probe assessing bi-spectral index (BIS; XP-sensor, Covidien plc, Dublin, Ireland) was also attached to the forehead to evaluate anesthesia depth. Subsequently, anesthesia was induced using sufentanil (0.1 µg/kg), propofol (1–1.5 mg/kg), and rocuronium (0.5 mg/kg). Following intubation, ventilation was set to 6–8 mL/kg ideal body weight, respiratory rate was adjusted to maintain etCO2 at 35–40 mmHg, and anesthesia was maintained using sevoflurane at BIS values between 40 and 60, ensuring an appropriate anesthesia level. Oxygen concentration was adjusted to maintain SpO_2_ above 95%. Next, a central line and sheath were inserted into the right jugular vein under ultrasound guidance. Surgery was then performed. At the conclusion of the procedure and following weaning from cardiopulmonary bypass (CPB), the team made a joint decision regarding the extubation strategy—either proceeding with extubation in the operating room (OR extubation, ORE) or transporting the patient intubated to the ICU for extubation within 6 h postoperatively (ICU extubation, ICUE). The decision for ORE was made on a case-by-case basis by the attending anesthesiologist in agreement with the surgical team after separation from cardiopulmonary bypass. Group allocation was not solely determined by the type of surgery, CPB duration, cross-clamp time, or comorbidities but rather on hemodynamic stability, bleeding complications, and respiratory and thermal stability. There were no absolute contraindications for an extubation attempt, but an extubation was never performed if anyone on the team was concerned about the patient's safety. For patients extubated in the OR within 1 h after surgery, the anesthesiologist who provided care during the case was responsible for postoperative extubation and transferred the spontaneously breathing patient to the ICU.

### 2.3 Statistical analyses

For descriptive statistics, continuous variables are reported as mean ± standard deviation (SD) or median (interquartile range, IQR), and categorical variables as counts and percentages. Patients were classified into ICU extubation (ICUE) and operating room extubation (ORE) groups. Propensity score matching (PSM) was conducted using logistic regression to reduce bias, with duration of cardiopulmonary bypass and EuroSCORE II as covariates. A one-to-one nearest-neighbor matching algorithm with a caliper of 0.2 was applied, resulting in 112 matched patients per group. We used the Wilcoxon signed-rank test for continuous variables and the McNemar test for nominal variables. Between-group differences for continuous variables are expressed as Hodges–Lehmann estimates of the pseudo median with 95% confidence intervals.

A significance level of 0.05 was used for all statistical tests without adjustment for multiple testing due to the exploratory nature of the trial. Statistical analyses were performed using the statistical programming environment R version 4.4.0 (Foundation for Statistical Computing, Vienna, Austria) using the MatchIt and cobalt add-on packages for matching and graphics (19).

## 3 Results

### 3.1 Cohort description and matching

A total of 397 patients undergoing elective on-pump cardiac surgery were included. Of these, 131 patients (33%) were extubated in the operating room (ORE) within 1 h after surgery, following the institutional ultrafast-track extubation (UFE) protocol. The remaining 266 patients (67%) were extubated upon ICU admission (ICUE), targeting extubation within 6 h postoperatively in accordance with the current guidelines (Figure 1). Preoperative and intraoperative characteristics were analyzed using the original, unmatched cohort, as these variables occur prior to extubation and are unaffected by the extubation strategy. This approach preserves the natural distribution of baseline characteristics and avoids introducing bias into variables used for propensity score matching, such as EuroSCORE II and cardiopulmonary bypass duration.

For the evaluation of postoperative outcomes, the matched cohort was used to ensure comparability between groups and to better isolate the impact of early (ORE) vs. delayed (ICUE) extubation on clinical endpoints. The primary endpoint of this study was hemodynamic stability assessed by the simplified acute physiology score (SAPS) and ICU workload measured by the Therapeutic Intervention Scoring System (TISS). Secondary endpoints comprised the total hospital and ICU length of stay (LOS), the duration of mechanical ventilation, and various clinical parameters, including catecholamine requirements.

### 3.2 Preoperative and intraoperative characteristics

#### 3.2.1 Demographics

There was no significant age (ORE 64 ± 11 years vs. ICUE 65 ± 10 years, mean ± SD) or height (ORE 173 ± 10 cm vs. ICUE 173 ± 11 cm, mean ± SD) difference between groups. However, ORE patients had significantly lower body weights (ORE 80 ± 17 kg vs. ICUE 87 ± 17 kg; mean ± SD; *p* < 0.001) and EuroSCORE [ORE 1.24 (1.11) vs. ICUE 1.89 (2.46); median (IQR); *p* < 0.001] (Table 1).

**Table 1:** Demographic and preoperative risk characteristics for the overall cohort (*N* = 397), patients extubated in the intensive care unit (ICUE, *n* = 266), and patients extubated in the operating room (ORE, *n* = 131) before propensity score matching.

Variable	Overall (*N* = 397)	ICUE (*N* = 266)	ORE (*N* = 131)	Difference (95% CI)	*p*-value
Age				2 (−4.85 × 10^−5^, 4)	0.114
Median (IQR)	66.0 (14.0)	66.5 (13.8)	64.0 (14.5)		
Mean (±SD)	64.6 (± 10.7)	65.1 (± 10.4)	63.5 (± 11.2)		
(Q1, Q3)	(58.0, 72.0)	(58.3, 72.0)	(57.0, 71.5)		
Weight				8 (4, 11)	<0.001
Median (IQR)	83.0 (22.0)	86.5 (22.8)	77.0 (21.0)		
Mean (±SD)	84.9 (± 17.7)	87.3 (± 17.4)	80.2 (± 17.3)		
(Q1, Q3)	(73.0, 95.0)	(75.0, 97.8)	(69.0, 90.0)		
Height				−5.61 × 10^−^^6^ (−2, 2)	0.944
Median (IQR)	174 (12.0)	174 (12.0)	176 (15.0)		
Mean (±SD)	173 (± 10.7)	173 (± 11.1)	173 (± 10.0)		
(Q1, Q3)	(168, 180)	(168, 180)	(165, 180)		
BMI				0.46 (0.24, 0.71)	<0.001
Mean (±SD)	28.4 (± 6.59)	29.3 (± 7.18)	26.5 (± 4.69)		
Median (IQR)	27.6 (6.86)	28.4 (6.47)	26.3 (6.16)		
(Q1, Q3)	(24.4, 31.3)	(25.2, 31.7)	(23.0, 29.1)		
EuroSCORE II				0.46 (0.24, 0.71)	<0.001
Median (IQR)	1.64 (2.04)	1.89 (2.46)	1.24 (1.11)		
Mean (±SD)	2.73 (± 3.94)	3.15 (± 4.55)	1.88 (± 1.97)		
(Q1, Q3)	(0.950, 2.99)	(1.07, 3.53)	(0.805, 1.91)		

Continuous variables are presented as median (interquartile range), mean ± standard deviation, and quartiles (Q1, Q3). Differences between ICUE and ORE are expressed as the Hodges–Lehmann estimate of the pseudomedian with 95% confidence intervals. *p*-values were obtained from the Wilcoxon rank-sum test. *p* < 0.05 was considered statistically significant.

#### 3.2.2 Procedural metrics

Heart–lung machine runs were notably shorter in the ORE group [ORE 1.45 (0.95) h vs. ICUE 2.20 (1.38) h, median (IQR), *p* < 0.001], as was the overall duration of surgery [ORE 2.33 (2.48) h vs. ICUE 4.78 (1.85) h, median (IQR), *p* < 0.001]. As a result of the protocol, mechanical ventilation duration was markedly reduced in ORE patients [ORE 4.42 (2.58) vs. ICUE 16.1 (9.17), median (IQR), *p* < 0.001] (Table 2).

**Table 2:** Procedural metrics for all patients, ICU extubation (ICUE) group, and operating room extubation (ORE) group in the unmatched cohort.

Variable	Overall (*N* = 397)	ICUE (*N* = 266)	ORE (*N* = 131)	Difference (95% CI)	*p*-value
Surgery (h)				1.98 (1.65, 2.32)	<0.001
Median (IQR)	4.32 (2.68)	4.78 (1.85)	2.33 (2.48)		
Mean (±SD)	4.26 (± 1.80)	4.88 (± 1.63)	3.01 (± 1.45)		
(Q1, Q3)	(2.71, 5.39)	(3.92, 5.77)	(1.78, 4.26)		
Missing	1 (0.3%)	1 (0.4%)			
On-pump (h)				0.7 (0.517, 0.883)	<0.001
Median (IQR)	1.93 (1.36)	2.20 (1.38)	1.45 (0.95)		
Mean (±SD)	2.13 (± 1.03)	2.39 (± 1.04)	1.61 (± 0.76)		
(Q1, Q3)	(1.33, 2.69)	(1.58, 2.97)	(1.04, 1.99)		
Missing	1 (0.3%)	1 (0.4%)			
Ventilation (h)				11 (9.87, 12.3)	<0.001
Median (IQR)	12.8 (13.1)	16.1 (9.17)	4.42 (2.58)		
Mean (±SD)	20.6 (± 41.2)	27.7 (± 48.6)	6.23 (± 7.45)		
(Q1, Q3)	(6.02, 19.1)	(12.4, 21.5)	(3.47, 6.04)		

Variables include total surgery duration and cardiopulmonary bypass (on-pump) time. Data are presented as median (interquartile range), mean ± standard deviation, and quartiles (Q1, Q3). Group differences are reported as Hodges–Lehmann estimates of the pseudomedian with 95% confidence intervals. *p*-values were obtained using the Wilcoxon rank-sum test. Missing data are indicated where applicable.

#### 3.2.3 Case severity and ICU labor

In the unmatched cohort, ICU workload and postoperative case severity were substantially lower in patients extubated in the operating room compared with those in patients extubated in the ICU. Median ICU length of stay was reduced by approximately 20 h in the ORE group [24.2 (45.0) h] compared with that in the ICUE group [49.4 (74.8) h; *p* < 0.001]. Postoperative TISS scores were markedly lower in the ORE group [median 9.0 (8.0)] than those in the ICUE group [19.0 (4.75)], with a pseudomedian difference of 10 points (95% CI: 10, 13; *p* < 0.001). A similar pattern was seen for postoperative SAPS scores [ORE 25.0 (13.0) vs. ICUE 34.0 (11.0); difference 8 points, 95% CI: 6, 10; *p* < 0.001]. Cumulative scores over the ICU stay further amplified these differences: cumulative TISS was 9.0 (16.5) in the ORE group vs. 38.0 (44.0) in the ICUE group (difference 24 points, 95% CI: 18, 29; *p* < 0.001), and cumulative SAPS was 30.0 (44.0) vs. 68.0 (105), respectively (difference 28 points, 95% CI 19, 39; *p* < 0.001) (Table 3).

**Table 3:** Hospitalization metrics, postoperative severity, and ICU workload for the unmatched cohort, comparing ICU extubation (ICUE) and operating room extubation (ORE) groups.

Variable	Overall (*N* = 397)	ICUE (*N* = 266)	ORE (*N* = 131)	Difference (95% CI)	*p*-value
ICU (h)				19.5 (7.8, 24.3)	<0.001
Median (IQR)	44.0 (68.3)	49.4 (74.8)	24.2 (45.0)		
Mean (±SD)	97.9 (± 197)	117 (± 226)	58.8 (± 110)		
(Q1, Q3)	(22.3, 90.6)	(23.7, 98.5)	(21.2, 66.2)		
Missing	1 (0.3%)	1 (0.4%)			
LOS (days)				2 (1, 3)	<0.001
Median (IQR)	11.0 (7.00)	12.0 (9.00)	10.0 (5.00)		
Mean (±SD)	14.7 (± 11.8)	16.1 (± 13.4)	12.0 (± 6.96)		
(Q1, Q3)	(9.00, 16.0)	(9.00, 18.0)	(8.00, 13.0)		
Missing	1 (0.3%)	1 (0.4%)			
TISS post-op				10 (10, 13)	<0.001
Median (IQR)	19.0 (9.00)	19.0 (4.75)	9.00 (8.00)		
Mean (±SD)	16.3 (± 7.17)	19.7 (± 5.03)	9.49 (± 5.92)		
(Q1, Q3)	(10.0, 19.0)	(18.3, 23.0)	(5.00, 13.0)		
SAPS post-op				8 (6, 10)	<0.001
Median (IQR)	31.0 (12.0)	34.0 (11.0)	25.0 (13.0)		
Mean (±SD)	31.5 (± 9.69)	34.0 (± 9.07)	26.4 (± 8.88)		
(Q1, Q3)	(25.0, 37.0)	(28.0, 39.0)	(20.0, 33.0)		
TISS cum				24 (18, 29)	<0.001
Median (IQR)	28.0 (39.0)	38.0 (44.0)	9.00 (16.5)		
Mean (±SD)	64.0 (± 150)	83.8 (± 177)	23.7 (± 51.3)		
(Q1, Q3)	(15.0, 54.0)	(19.0, 63.0)	(5.00, 21.5)		
SAPS cum				28 (19, 39)	<0.001
Median (IQR)	53.0 (80.0)	68.0 (105)	30.0 (44.0)		
Mean (±SD)	134 (± 297)	168 (± 347)	64.9 (± 131)		
(Q1, Q3)	(30.0, 110)	(37.0, 142)	(22.0, 66.0)		

Metrics include ICU length of stay (hours), total hospital length of stay (days), and severity/resource utilization scores (Therapeutic Intervention Scoring System, TISS; simplified acute physiology score, SAPS), assessed both postoperatively and cumulatively over the ICU stay. Values are reported as median (interquartile range), mean ± standard deviation, and quartiles (Q1, Q3). Group differences are expressed as Hodges–Lehmann estimates with 95% confidence intervals, and *p*-values are from Wilcoxon rank-sum tests. *p* < 0.05 was considered statistically significant; missing data are indicated where applicable.

#### 3.2.4 Hemodynamic and transfusion parameters

The vasoactive–inotropic score (VIS) was calculated to compare the weight sum of all administered vasopressors and inotropic medications. After induction of anesthesia and before incision, pharmacological cardiovascular therapy was not different between groups [ORE 4.00 (4.00) vs. ICUE 3.00 (4.00), median (IQR), *p* = 0.615]. Postoperatively, VIS scores were significantly lower in the ORE group compared with those in patients who were mechanically ventilated during transport to the ICU and extubated within 6 h upon admission [ORE 2.00 (4.12) vs. ICUE 8.14 (20.1), median (IQR), *p* < 0.001]. Furthermore, patients extubated during ICU treatment required vasoactive substances for a longer duration compared with patients extubated in the OR (Table 4).

**Table 4:** Hemodynamic support and transfusion parameters in the unmatched cohort, comparing ICU extubation (ICUE) and operating room extubation (ORE) groups.

Variable	Overall (*N* = 397)	ICUE (*N* = 266)	ORE (*N* = 131)	Difference (95% CI)	*p*-value
VIS pre-op				NA	0.615
Median (IQR)	3.17 (4.00)	3.00 (4.00)	4.00 (4.00)		
Mean (±SD)	5.62 (± 16.7)	6.12 (± 20.3)	4.60 (± 3.98)		
(Q1, Q3)	(2.00, 6.00)	(2.00, 6.00)	(2.00, 6.00)		
VIS post-op				NA	<0.001
Median (IQR)	5.15 (12.3)	8.14 (20.1)	2.00 (4.12)		
Mean (±SD)	43.0 (± 116)	59.9 (± 136)	8.81 (± 38.8)		
(Q1, Q3)	(2.00, 14.3)	(3.19, 23.3)	(0, 4.12)		
RBC transfusion OR (mL)				3.38 × 10^−^^5^ (8.6 × 10^−^^5^, 3.64 × 10^−^^5^)	<0.001
Median (IQR)	0 (250)	0 (500)	0 (0)		
Mean (±SD)	227 (± 445)	305 (± 517)	69.7 (± 142)		
(Q1, Q3)	(0, 250)	(0, 500)	(0, 0)		
RBC transfusion sum post-op (mL)				1.93 × 10^−^^5^ (−1.28 × 10^−^^5^, 4.35 × 10^−^^5^)	0.2
Median (IQR)	250 (500)	250 (750)	250 (500)		
Mean (±SD)	433 (± 685)	435 (± 642)	427 (± 769)		
(Q1, Q3)	(0, 500)	(0, 750)	(0, 500)		

Vasoactive–inotropic score (VIS) was measured preoperatively and postoperatively to quantify pharmacologic cardiovascular support. Red blood cell (RBC) transfusion volumes are presented for intraoperative administration and cumulative postoperative totals. Data are expressed as median (interquartile range), mean ± standard deviation, and quartiles (Q1, Q3). Group differences are shown as Hodges–Lehmann estimates of the pseudomedian with 95% confidence intervals (NA when not calculable). Statistical comparisons used the Wilcoxon rank-sum test, with *p* < 0.05 considered significant.

### 3.3 Propensity score matching and covariate balance

After propensity score matching, cohorts were comparable in terms of age (ICU 63.4 ± 10.8 years vs. ORE 63.6 ± 10.7 years) and height (174 ± 11.1 cm vs. 174 ± 10.1 cm). However, the ORE group had a lower mean weight (80.0 ± 17.2 kg) compared with the ICUE group (89.7 ± 17.6 kg). Baseline hemoglobin concentration levels (14.1 ± 1.81 vs. 13.7 ± 1.26 g/dl) were comparable between groups (Supplementary Table S1).

#### 3.3.1 Preoperative hemodynamic and vasopressor support

Baseline vasopressor and inotropic support showed no significant differences between groups, with comparable preoperative vasoactive–inotropic scores (ICUE: 5.48 ± 16.5 vs. ORE: 4.79 ± 3.88). However, the preoperative use of milrinone was observed only in the ICUE group (0.155 ± 1.64 µg/kg/h), while none in the ORE group received this medication. Preoperative norepinephrine (ICUE: 0.0365 ± 0.0284 µg/kg/min vs. ORE: 0.0447 ± 0.0378 µg/kg/min) and dobutamine doses (ICU: 0.240 ± 0.897 µg/kg/min vs. ORE: 0.316 ± 0.948 µg/kg/min) were comparable between groups (Supplementary Table S1). Complications, including revision, sepsis pneumonia, ECMO, wound infection, delirium, seizure, and reintubation rates, were not different between groups (Supplementary Table S3).

#### 3.3.2 Postoperative outcomes after propensity score matching

The matched analysis confirmed the results observed in the unmatched data results. Upon ICU admission, the ORE group continued to demonstrate significantly lower TISS scores (9.62 ± 6.13 vs. 19.6 ± 5.25; *p* < 0.001) and SAPS scores (26.3 ± 8.92 vs. 32.1 ± 8.50; *p* < 0.001). Cumulative ICU workload and illness severity remained markedly lower in the ORE group, with cumulative TISS scores of 25.3 ± 55.2 vs. 55.3 ± 69.2 (*p* < 0.001) and cumulative SAPS scores of 69.0 ± 141 vs. 109 ± 161 (*p* < 0.001).

In addition, matched analysis showed that patients extubated in the OR had a substantially shorter duration of mechanical ventilation (6.65 ± 7.98 h vs. 23.8 ± 39.7 h; *p* < 0.001) and shorter ICU stay (63.1 ± 118 h vs. 82.2 ± 119 h; *p* = 0.023), while total hospital length of stay (LOS) did not differ significantly between groups (12.3 ± 7.39 days vs. 13.2 ± 9.44 days; *p* = 0.432) (Table 5).

**Table 5:** Duration of mechanical ventilation, length of stay, severity of illness, and ICU workload in the propensity score-matched cohort comparing ICU extubation (ICUE) and operating room extubation (ORE) groups.

Variable	Overall (*N* = 224)	ICUE (*N* = 112)	ORE (*N* = 112)	Difference (95% CI)	*p*-value
Ventilation (h)				−9.03 (−10.7, −7.81)	<0.001
Median (IQR)	8.97 (10.1)	13.7 (7.23)	4.68 (2.61)		
Mean (±SD)	15.2 (± 29.9)	23.8 (± 39.7)	6.65 (± 7.98)		
(Q1, Q3)	(4.57, 14.7)	(11.4, 18.6)	(3.69, 6.30)		
ICU (h)				−12.4 (−24.2, −1.75)	0.023
Median (IQR)	28.3 (49.9)	45.0 (55.5)	24.5 (48.2)		
Mean (±SD)	72.7 (± 119)	82.2 (± 119)	63.1 (± 118)		
(Q1, Q3)	(21.5, 71.4)	(22.5, 78.0)	(21.3, 69.5)		
LOS (days)				−0.5 (−2, 1)	0.432
Median (IQR)	10.5 (6.00)	11.0 (5.00)	10.0 (6.00)		
Mean (±SD)	12.7 (± 8.47)	13.2 (± 9.44)	12.3 (± 7.39)		
(Q1, Q3)	(8.00, 14.0)	(9.00, 14.0)	(8.00, 14.0)		
TISS post-op				−11.5 (−12, −9.5)	<0.001
Median (IQR)	15.0 (10.0)	19.0 (4.00)	9.00 (8.25)		
Mean (±SD)	14.6 (± 7.59)	19.6 (± 5.25)	9.62 (± 6.13)		
(Q1, Q3)	(9.00, 19.0)	(19.0, 23.0)	(5.00, 13.3)		
SAPS post-op				−6 (−8.5, −4)	<0.001
Median (IQR)	28.0 (13.0)	31.5 (9.25)	25.0 (12.3)		
Mean (±SD)	29.2 (± 9.16)	32.1 (± 8.50)	26.3 (± 8.92)		
(Q1, Q3)	(23.0, 36.0)	(26.8, 36.0)	(20.0, 32.3)		
TISS cum				−23.5 (−30.5, −17)	<0.001
Median (IQR)	19.0 (37.0)	37.5 (35.0)	10.0 (18.0)		
Mean (±SD)	40.3 (± 64.2)	55.3 (± 69.2)	25.3 (± 55.2)		
(Q1, Q3)	(9.00, 46.0)	(19.0, 54.0)	(5.00, 23.0)		
SAPS cum				−25.5 (−40.5, −13)	<0.001
Median (IQR)	39.5 (60.0)	57.5 (65.8)	30.0 (47.3)		
Mean (±SD)	89.2 (± 152)	109 (± 161)	69.0 (± 141)		
(Q1, Q3)	(25.0, 85.0)	(31.8, 97.5)	(22.0, 69.3)		

Variables include mechanical ventilation time (hours), ICU stay (hours), total hospital stay (LOS, days), and severity/resource utilization scores (Therapeutic Intervention Scoring System, TISS; simplified acute physiology score, SAPS), reported both postoperatively and cumulatively for the ICU stay. Data are presented as median (interquartile range), mean ± standard deviation, and quartiles (Q1, Q3). Group differences are expressed as Hodges–Lehmann estimates of the pseudomedian with 95% confidence intervals. *p*-values are from Wilcoxon rank-sum tests, with *p* < 0.05 considered statistically significant.

#### 3.3.3 Hemodynamic and intensive care parameters

The overall duration of catecholamine support was significantly longer in the ICUE group compared with that in the ORE group [median (IQR): 42.2 (52.1) vs. 26.9 (38.3) h; *p* < 0.005]. Additionally, the required dosages of vasoactive agents were consistently higher in ICUE patients compared with those in ORE patients. Initial postoperative dobutamine requirements were significantly greater in the ICU group than those in the ORE group (1.99 ± 1.82 vs. 1.41 ± 1.44 µg/kg/min; *p* < 0.01), with this difference persisting at 48 h postoperatively (0.648 ± 1.36 vs. 0.296 ± 0.991 µg/kg/min; *p* < 0.05). Similarly, norepinephrine usage was significantly higher in the ICUE group (0.107 ± 0.270 vs. 0.0222 ± 0.0461 µg/kg/min; *p* < 0.001). Milrinone requirements were also greater in ICUE patients compared with those in the ORE group (2.27 ± 6.81 vs. 0.628 ± 4.12 µg/kg/h; *p* = 0.005). The vasoactive–inotropic score (VIS) was significantly higher in the ICUE group at the end of the surgery [7.00 (12.0) vs. 2.16 (3.39); *p* < 0.001] and remained elevated at 12 and 24 h (*p* < 0.05), indicating a sustained need for cardiovascular support in these patients (Supplementary Table S2).

#### 3.3.4 Case severity and ICU labor

**Figure 2:**
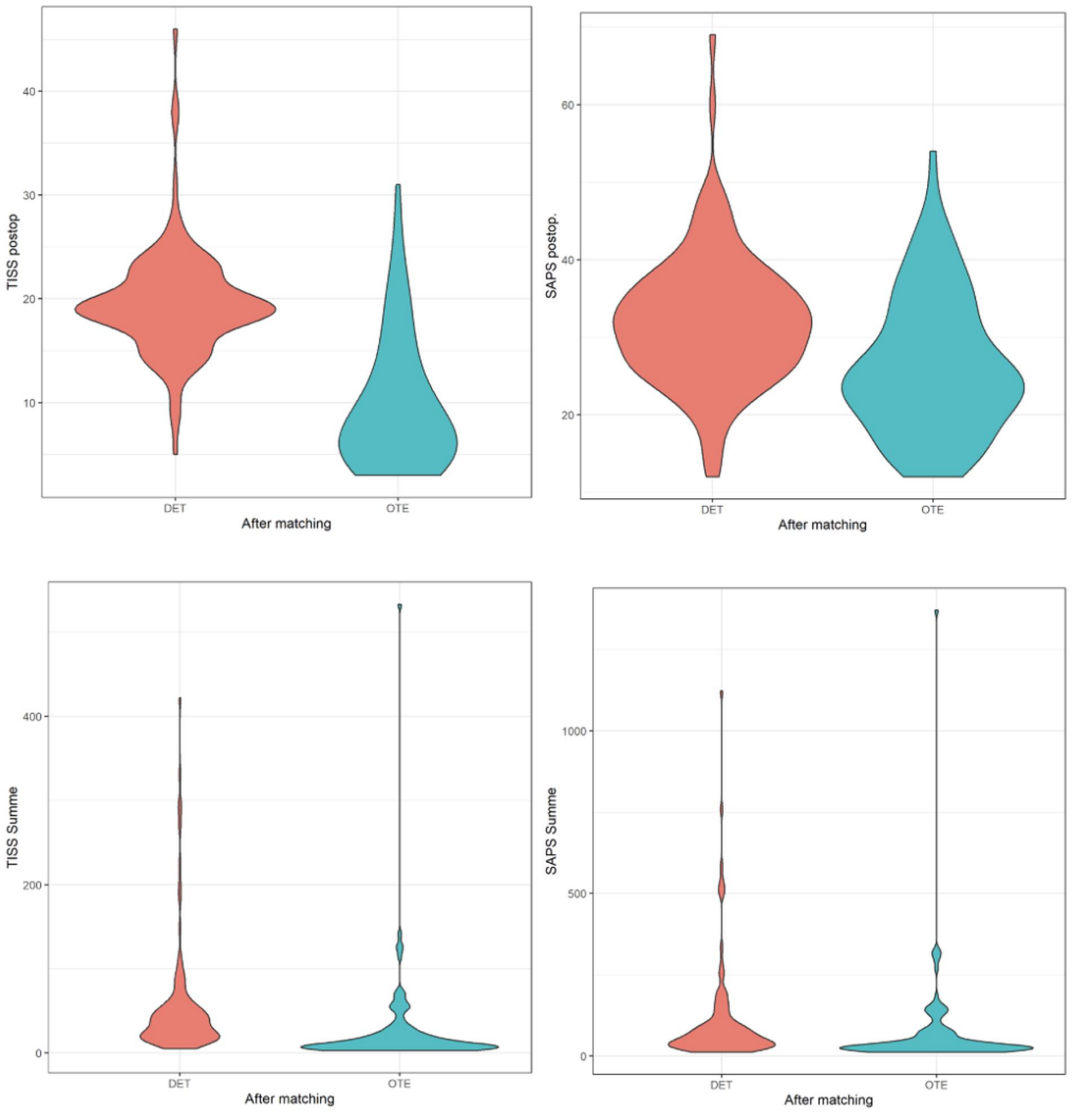
Violin plots of Therapeutic Intervention Scoring System (TISS) and simplified acute physiology score (SAPS) values between patients extubated in the ICU (DET) and those extubated in the operating room (OTE), following propensity score matching. The top row shows postoperative TISS and postoperative SAPS values. The bottom row displays cumulative TISS and cumulative SAPS scores over the ICU stay. Violin plots illustrate the distribution, median, and density of each variable, highlighting reduced severity and ICU workload in the OTE group compared with the DET group.

TISS scores, reflecting therapeutic workload, were significantly higher in ICUE patients compared with those in ORE patients both on the day of surgery (19.6 ± 5.25 vs. 9.62 ± 6.13; *p* < 0.001) and cumulatively over the ICU stay (55.3 ± 69.2 vs. 25.3 ± 55.2; *p* < 0.001). Similarly, SAPS scores, indicating physiological severity, were significantly higher in the ICUE group compared with those in the ORE group on the day of surgery (32.1 ± 8.50 vs. 26.3 ± 8.92; *p* < 0.001) and cumulatively (109 ± 161 vs. 69.0 ± 141; *p* < 0.001). These findings highlight that patients extubated in the ICU required substantially greater therapeutic interventions and presented with higher case severity, underscoring one of the central observations of this study (Figure 2).

### 3.4 Summary of key findings

The ICUE group demonstrated a higher need for catecholamine support, longer ICU stays, and increased postoperative severity scores. However, major postoperative complications, transfusion requirements, and overall hospital stay did not differ significantly between groups. The findings suggest that while ICUE is associated with increased postoperative resource utilization, its impact on overall patient outcomes warrants further investigation. Overall, despite a similar baseline risk profile, the ORE group had shorter operative times, lower intraoperative transfusion requirements, and a similar need for preoperative hemodynamic support compared with the DET group.

## 4 Discussion

The findings of this study reveal that ultrafast extubation (UFE) in the operating room within 1 h after cardiac surgery is not only clinically safe and contributes to reduced ICU workload and shorter treatment duration. Our results suggest that patients undergoing UFE significantly benefit from improved physiological stability, indicated by decreased SAPS scores and catecholamine requirements, leading to significantly reduced ICU length of stay, finally resulting in reduced ICU workload as indicated by lower TISS scores. Collectively, these improvements directly translate to cost savings and more efficient utilization of limited intensive care capacity—a key constraint in modern healthcare systems. These findings are in accordance with the growing body of evidence supporting early extubation strategies mainly in designated ERACS areas and within 6 h after surgery but show an earlier extubation being even more beneficial than the current recommended standard of care (12, 13).

Reduced ICU workload has several important implications. First, shorter ICU treatment durations directly reduce operational strain and staffing demands, particularly critical in settings suffering from nursing shortages, as experienced overall in the post-COVID area. Second, improved ICU throughput enhances access to surgical care for subsequent patients, reducing delays of scheduled procedures, mitigating the negative consequences of postponed interventions, particularly in the context of cardiac and tumor surgeries. Such cascade effects not only improve institutional efficiency but also elevate overall patient outcomes by enabling timely care.

Importantly, the reduced need for vasopressors and red blood cell (RBC) transfusions in the ORE group reflects a more stable postoperative course with fewer complications and a decreased requirement for intensive monitoring and interventions. The lower rate of red blood cell transfusions observed in the ORE group may be attributed to hemodilution resulting from greater fluid administration in the ICUE group, which could have led to a more pronounced reduction in hematocrit levels. However, this is a critical finding given the well-established association between perioperative blood transfusions and adverse outcomes, including increased infection risk and prolonged recovery (14, 15). These results are consistent with prior studies demonstrating improved hemodynamic stability, lower inflammatory burden, and reduced delirium with early extubation protocols (16). We have not observed lower delirium rates after propensity score matching. Nevertheless, reduced sedation exposure and earlier mobilization in the UFE group remain plausible contributors to improved neurocognitive outcomes. A potential explanation for this is the overall low incidence of delirium in ICUE patients compared with the overall published incidence, indicative of overall good perioperative management. However, reduced delirium rates could be attributed to decreased exposure to sedative agents, earlier mobilization, and improved sleep–wake cycles, all of which have been implicated in delirium prevention strategies (17). Moreover, the comparable rates of reintubation and major complications reinforce that these resource savings are not achieved at the cost of patient safety, further underlining the safety of the UFE approach.

Economic analyses in similar contexts have shown that even modest reductions in ICU days potentially yield substantial savings, particularly in high-cost environments such as cardiac surgery and corresponding intensive care medicine. For example, a study by Evans et al. (18) reported a reduction of 24 h per patient can equate to thousands of dollars in healthcare expenses saved per case, depending on the local healthcare model. Similarly, implementing enhanced recovery after surgery protocols in cardiac surgery (ERACS) has been associated with significant reductions in ICU and hospital stays, leading to cost savings of approximately €1,900 per patient (15). Our data, which demonstrate a reduction of nearly 50% in ICU time, highlight the scalability and system-level benefit of integrating UFE protocols as standard practice. These findings underscore the potential economic benefits of strategies aimed at decreasing ICU utilization in cardiac surgical care.

Finally, from a healthcare systems perspective, the implementation of UFE contributes to cost-effectiveness not only by reducing direct ICU costs but also by expanding ICU availability. This strategy enables more procedures to be completed within existing capacities, potentially reducing patient wait times, avoiding disease progression, and increasing surgical volume—all of which contribute to better outcomes and financial sustainability.

Despite these promising findings, several limitations warrant consideration. First, the retrospective single-center design limits external validity and carries the potential for selection bias, particularly regarding the intraoperative decision to extubate early. We acknowledge that patients judged suitable for early extubation may have had more favorable intraoperative courses or baseline characteristics. To mitigate this, we applied rigorous propensity score matching using a broad set of relevant pre-, intra-, and postoperative variables, including the EuroSCORE II, which—while not perfect—remains a widely accepted measure of operative risk in cardiac surgery. This approach achieved a high degree of balance between groups, reducing but not fully eliminating the possibility of residual confounding from unmeasured factors such as frailty, subtle intraoperative events, or team-specific preferences. Second, long-term endpoints, including 30-day mortality, readmissions, or patient-centered quality-of-life measures, were not assessed. Third, the generalizability of these findings to centers with dedicated post anesthesia care unit (PACUs), alternative perioperative pathways, or different staffing models requires further validation. Moreover, while the propensity score matching approach effectively balanced observed baseline characteristics, residual confounding from unmeasured variables—such as team-specific extubation decision-making preferences or patient frailty—cannot be excluded and should be considered when interpreting these findings.

## 5 Conclusions

In conclusion, the results support that ultrafast extubation after cardiac surgery improves hemodynamic stability and reduces ICU workload, potentially reducing healthcare expenses. Future research should focus on long-term outcome data and broader implementation strategies across institutions to validate its generalizability and economic benefit.

## Data Availability

The datasets generated and analyzed during the current study are not publicly available due to institutional and regulatory restrictions related to patient confidentiality and data protection policies (HIPAA and institutional IRB requirements). De-identified data may be made available from the corresponding author upon reasonable request and with permission from the relevant institutional review boards.
