# Supplementary material for: Ultra-fast extubation following cardiac surgery improves hemodynamic stability and reduces ICU workload
Source: Front Cardiovasc Med. 2025 Nov 24;12:1695955. doi: 10.3389/fcvm.2025.1695955 (PMC12682788; doi:10.3389/fcvm.2025.1695955)
Supplement: Supplementary file 1 [file Datasheet1.pdf]

Supplementary Material

Table S1

Variable	Overall (N=224)	ICUE (N=112)	ORE (N=112)
**Age**			
Median [IQR]	64.0 [15.0]	64.5 [15.0]	63.0 [14.5]
Mean (±SD)	63.5 (± 10.7)	63.4 (± 10.8)	63.6 (± 10.7)
**Height**			
Median [IQR]	176 [12.0]	175 [12.0]	176 [15.3]
Mean (±SD)	174 (± 10.6)	174 (± 11.1)	174 (± 10.1)
**Hb preOP**			
Median [IQR]	13.9 [1.90]	14.3 [2.10]	13.6 [1.6]
Mean (±SD)	13.9 (± 1.57)	14.1 (± 1.81)	13.7 (± 1.26)
**Weight**			
Median [IQR]	82.0 [24.0]	89.0 [21.3]	76.5 [21.3]
Mean (±SD)	84.9 (± 18.0)	89.7 (± 17.6)	80.0 (± 17.2)
**VIS**			
Median [IQR]	4.00 [4.00]	3.00 [3.25]	4.00 [4.00]
Mean (±SD)	5.13 (± 12.0)	5.48 (± 16.5)	4.79 (± 3.88)
**Milrinone**			
Median [IQR]	0 [0]	0 [0]	0 [0]
Mean (±SD)	0.078 (± 1.16)	0.155 (± 1.64)	0 (± 0)
**norepinehrine**			
Median [IQR]	0.03 [0.03]	0.03 [0.03]	0.04 [0.03]
Mean (±SD)	0.041 (± 0.03)	0.037 (± 0.028)	0.045 (± 0.038)
**dobutamine**			
Median [IQR]	0 [0]	0 [0]	0 [0]
Mean (±SD)	0.278 (± 0.922)	0.240 (± 0.897)	0.316 (± 0.948)

Table S1: Preoperative Characteristics After Propensity Score Matching: Key preoperative variables of the overall cohort (N=224) and matched groups undergoing either intracardiac ultrasound-guided extubation (ICUE, N=112) or operating room extubation (ORE, N=112). Data are presented as median with interquartile range [IQR] and mean with standard deviation (±SD), as appropriate. Variables include demographic measures (age, height, weight), preoperative hemoglobin (Hb preOP), vasoactive inotropic score (VIS), and preoperative vasoactive medication dosages (milrinone, norepinephrine, dobutamine). Matching resulted in balanced baseline characteristics between groups.

Table S2

**Post_Dobutamin (µg/kg/min)**				Wilcoxon	0.0088
Median [IQR]	1.76 [2.66]	2.00 [2.75]	1.34 [2.44]		
Mean (±SD)	1.70 (± 1.67)	1.99 (± 1.82)	1.41 (± 1.44)		
**12h_Dobutamin (µg/kg/min)**				Wilcoxon	0.31
Median [IQR]	0.970 [2.21]	1.15 [2.20]	0 [2.22]		
Mean (±SD)	1.29 (± 1.62)	1.38 (± 1.55)	1.20 (± 1.69)		
**24h_Dobutamin (µg/kg/min)**				Wilcoxon	0.684
Median [IQR]	0 [1.25]	0 [1.89]	0 [0]		
Mean (±SD)	0.818 (± 1.46)	0.853 (± 1.27)	0.782 (± 1.64)		
**48h_Dobutamin (µg/kg/min)**				Wilcoxon	0.0291
Median [IQR]	0 [0]	0 [1.00]	0 [0]		
Mean (±SD)	0.472 (± 1.20)	0.648 (± 1.36)	0.296 (± 0.991)		
**72h_Dobutamin (µg/kg/min)**				Wilcoxon	0.247
Median [IQR]	0 [0]	0 [0]	0 [0]		
Mean (±SD)	0.264 (± 0.881)	0.357 (± 1.02)	0.171 (± 0.707)		
**Post_Suprarenin (µg/kg/min)**				Wilcoxon	0.0733
Median [IQR]	0 [0]	0 [0]	0 [0]		
Mean (±SD)	0.00411 (± 0.0183)	0.00598 (± 0.0196)	0.00223 (± 0.0167)		
**12h_Suprarenin (µg/kg/min)**				Wilcoxon	0.31
Median [IQR]	0 [0]	0 [0]	0 [0]		
Mean (±SD)	0.0176 (± 0.228)	0.0335 (± 0.322)	0.00179 (± 0.0156)		
**24h_Suprarenin (µg/kg/min)**				Wilcoxon	0.735
Median [IQR]	0 [0]	0 [0]	0 [0]		
Mean (±SD)	0.00433 (± 0.0369)	0.00330 (± 0.0195)	0.00536 (± 0.0485)		
**48h_Suprarenin (µg/kg/min)**				Wilcoxon	0.833
Median [IQR]	0 [0]	0 [0]	0 [0]		
Mean (±SD)	0.00143 (± 0.00964)	0.00170 (± 0.0116)	0.00116 (± 0.00720)		
**72h_Suprarenin (µg/kg/min)**				Wilcoxon	0.584
Median [IQR]	0 [0]	0 [0]	0 [0]		
Mean (±SD)	0.000982 (± 0.00745)	0.00116 (± 0.00867)	0.000804 (± 0.00602)		

**Post_Arterenol (µg/kg/min)**				Wilcoxon	9.71e-10
Median [IQR]	0.0200 [0.0700]	0.0400 [0.0900]	0 [0.0200]		
Mean (±SD)	0.0645 (± 0.198)	0.107 (± 0.270)	0.0222 (± 0.0461)		
**12h_Arterenol (µg/kg/min)**				Wilcoxon	0.0866
Median [IQR]	0 [0.0600]	0 [0.0900]	0 [0.0400]		
Mean (±SD)	0.0571 (± 0.123)	0.0687 (± 0.129)	0.0456 (± 0.115)		
**24h_Arterenol (µg/kg/min)**				Wilcoxon	0.065
Median [IQR]	0 [0.0100]	0 [0.0300]	0 [0]		
Mean (±SD)	0.0243 (± 0.0545)	0.0300 (± 0.0595)	0.0186 (± 0.0486)		
**48h_Arterenol (µg/kg/min)**				Wilcoxon	0.739
Median [IQR]	0 [0]	0 [0]	0 [0]		
Mean (±SD)	0.0180 (± 0.0678)	0.0214 (± 0.0859)	0.0146 (± 0.0428)		
**72h_Arterenol (µg/kg/min)**				Wilcoxon	0.983
Median [IQR]	0 [0]	0 [0]	0 [0]		
Mean (±SD)	0.0107 (± 0.0623)	0.0140 (± 0.0817)	0.00741 (± 0.0333)		
**Post_Milrinon (Corotrop) (µg/kg/h)**				Wilcoxon	0.00536
Median [IQR]	0 [0]	0 [0]	0 [0]		
Mean (±SD)	1.45 (± 5.68)	2.27 (± 6.81)	0.628 (± 4.12)		
**12h_Milrinon (Corotrop) (µg/kg/h)**				Wilcoxon	0.00695
Median [IQR]	0 [0]	0 [0]	0 [0]		
Mean (±SD)	1.02 (± 4.54)	1.82 (± 6.11)	0.218 (± 1.65)		
**24h_Milrinon (Corotrop) (µg/kg/h)**				Wilcoxon	0.0806
Median [IQR]	0 [0]	0 [0]	0 [0]		
Mean (±SD)	0.985 (± 4.42)	1.51 (± 5.44)	0.459 (± 3.02)		
**48h_Milrinon (Corotrop) (µg/kg/h)**				Wilcoxon	0.415
Median [IQR]	0 [0]	0 [0]	0 [0]		
Mean (±SD)	0.878 (± 4.64)	1.12 (± 5.19)	0.638 (± 4.02)		
**72h_Milrinon (Corotrop) (µg/kg/h)**				Wilcoxon	0.236
Median [IQR]	0 [0]	0 [0]	0 [0]		
Mean (±SD)	0.656 (± 3.73)	0.985 (± 4.64)	0.327 (± 2.49)		
**Post_Vasopressin (U/kg/h)**				Wilcoxon	0.371
Median [IQR]	0 [0]	0 [0]	0 [0]		

Mean (±SD)	0.000134 (± 0.00149)	0.000268 (± 0.00211)	0 (± 0)		
**12h_Vasopressin (U/kg/h)**				Wilcoxon	0.345
Median [IQR]	0 [0]	0 [0]	0 [0]		
Mean (±SD)	0.000313 (± 0.00290)	0.000536 (± 0.00399)	0.0000893 (± 0.000945)		
**24h_Vasopressin (U/kg/min)**				Wilcoxon	1
Median [IQR]	0 [0]	0 [0]	0 [0]		
Mean (±SD)	0.000179 (± 0.00267)	0.000357 (± 0.00378)	0 (± 0)		
**48h_Vasopressin (U/kg/h)**				Wilcoxon	1
Median [IQR]	0 [0]	0 [0]	0 [0]		
Mean (±SD)	0.000107 (± 0.00115)	0.0000893 (± 0.000945)	0.000125 (± 0.00132)		
**72_Vasopressin (U/kg/min)**				Wilcoxon	0.371
Median [IQR]	0 [0]	0 [0]	0 [0]		
Mean (±SD)	0.000134 (± 0.00149)	0.000268 (± 0.00211)	0 (± 0)		
**VIS postop.**				Wilcoxon	5.23e-09
Median [IQR]	3.86 [9.14]	7.00 [12.0]	2.16 [3.39]		
Mean (±SD)	24.4 (± 75.4)	38.7 (± 96.1)	10.1 (± 41.9)		
**VIS 12h**				Wilcoxon	0.0167
Median [IQR]	2.16 [9.40]	2.52 [13.2]	1.31 [6.11]		
Mean (±SD)	22.1 (± 75.4)	35.2 (± 102)	9.01 (± 24.7)		
**VIS 24h**				Wilcoxon	0.0449
Median [IQR]	0 [4.00]	0.500 [5.40]	0 [2.96]		
Mean (±SD)	15.3 (± 66.6)	22.9 (± 87.9)	7.77 (± 32.6)		
**VIS 48h**				Wilcoxon	0.463
Median [IQR]	0 [1.71]	0 [2.14]	0 [0]		
Mean (±SD)	12.3 (± 60.4)	15.0 (± 67.7)	9.49 (± 52.4)		
**VIS 72h**				Wilcoxon	0.388
Median [IQR]	0 [0]	0 [0]	0 [0]		
Mean (±SD)	9.33 (± 52.2)	14.4 (± 68.9)	4.26 (± 25.9)		

Table S2: Postoperative Vasoactive Medication Use and Vasoactive-Inotropic Score (VIS)—including dobutamine, suprarenin (epinephrine), arterenol (norepinephrine), milrinone, and vasopressin—measured in µg/kg/min, µg/kg/h, or U/kg/h at timepoints immediately postoperatively and at 12, 24, 48, and 72 hours. The vasoactive-inotropic score (VIS) was calculated at the same time intervals. Data are shown for the overall matched cohort (N=224) and stratified into the intracardiac ultrasound-guided extubation group (ICUE, N=112) and the operating room extubation group (ORE, N=112).Values are reported as median with interquartile range [IQR] and mean with

standard deviation (±SD). Between-group comparisons were performed using the Wilcoxon rank-sum test.

Table S3.

Variable	Overall (N=224)	ICUE (N=112)	ORE (N=112)		
**Complications**				McNemar	0.377
Yes	38 (17 %)	22 (20 %)	16 (14 %)		
No	186 (83 %)	90 (80 %)	96 (86 %)		
**Revision**				McNemar	1
Yes	24 (11 %)	12 (11 %)	12 (11 %)		
No	200 (89 %)	100 (89 %)	100 (89 %)		
**Sepsis**				McNemar	1
Yes	1 (0 %)	1 (1 %)	0 (0 %)		
No	223 (100 %)	111 (99 %)	112 (100 %)		
**Pneumonia**				McNemar	0.134
Yes	4 (2 %)	4 (4 %)	0 (0 %)		
No	220 (98 %)	108 (96 %)	112 (100 %)		
**ECMO**				McNemar	1
Yes	1 (0 %)	1 (1 %)	0 (0 %)		
No	223 (100 %)	111 (99 %)	112 (100 %)		
**Wound infection**				McNemar	1
Yes	2 (1 %)	1 (1 %)	1 (1 %)		
No	222 (99 %)	111 (99 %)	111 (99 %)		
**Delirium**				McNemar	0.683
Yes	6 (3 %)	4 (4 %)	2 (2 %)		
No	218 (97 %)	108 (96 %)	110 (98 %)		
**Seizure**				McNemar	1
Yes	4 (2 %)	2 (2 %)	2 (2 %)		
No	220 (98 %)	110 (98 %)	110 (98 %)		
**Reintubation**				McNemar	0.502
Yes	22 (10 %)	9 (8 %)	13 (12 %)		
No	202 (90 %)	103 (92 %)	99 (88 %)		

Table S3: Postoperative complications including Data are shown for the overall matched cohort (N=224) and stratified into the intracardiac ultrasound-guided extubation group (ICUE, N=112) and the operating room extubation group (ORE, N=112). Between-group comparisons were performed using the McNemar test.
